# Artificial intelligence-driven shimming for parallel high field nuclear magnetic resonance

**DOI:** 10.1038/s41598-023-45021-6

**Published:** 2023-10-20

**Authors:** Moritz Becker, Yen-Tse Cheng, Achim Voigt, Ajmal Chenakkara, Mengjia He, Sören Lehmkuhl, Mazin Jouda, Jan G. Korvink

**Affiliations:** Institute of Microstructure Technology (IMT), Karlsruhe Institute of Technology (KIT), Eggenstein-Leopoldshafen, 76344 Karlsruhe, Germany

**Keywords:** Electrical and electronic engineering, Solution-state NMR, Solution-state NMR

## Abstract

Rapid drug development requires a high throughput screening technology. NMR could benefit from parallel detection but is hampered by technical obstacles. Detection sites must be magnetically shimmed to ppb uniformity, which for parallel detection is precluded by commercial shimming technology. Here we show that, by centering a separate shim system over each detector and employing deep learning to cope with overlapping non-orthogonal shimming fields, parallel detectors can be rapidly calibrated. Our implementation also reports the smallest NMR stripline detectors to date, based on an origami technique, facilitating further upscaling in the number of detection sites within the magnet bore.

## Introduction

Fast drug discovery from a large cohort of candidate ligands, such as required for the rapid development of vaccines, demands a high throughput screening option to achieve rapid results within days or weeks. Nuclear magnetic resonance (NMR) spectroscopy is a suitable screening method, and a powerful technique for characterizing chemical ligands, compounds, and biologically active molecules, while being non-destructive and non-invasive compared to other standard methods such as mass spectrometry or X-ray crystallography. However, conventional NMR is limited by low throughput, and requires time-consuming steps such as sample loading, coil tuning, and magnetic field shimming, before a useful measurement is obtained. Recent developments aim for faster experiments, e.g., through the use of sample flow arrangements^1^ as opposed to repeated loading of discrete sample tubes, or sample hyperpolarization with around $100\times$ signal enhancement^2^. However, most (expensive) superconducting magnet bores are equipped with a single detection coil and perform experiments sequentially.

To overcome these challenges, parallel NMR spectroscopy has emerged as a promising approach that can simultaneously acquire spectra from multiple samples or regions of interest, speeding up the time of measurement^3–5^. However, parallel NMR also faces technical challenges, such as handling multiple samples, achieving sufficiently high spectral resolution, and allowing independent unperturbed operation of the parallel detector sites. These difficulties can be tackled by a multiple-coil system, based on an integrated NMR cell (NC) arrangement^3^. In this paper, we adapt the concept of an NC to operation within a $15.2\,T$ superconducting magnetic resonance imaging (MRI) magnet, to benefit from the linear increase of the chemical shift line separation with $B_0$ field strength, and a sensitivity increase that scales with $B_0^{7/4}$^6^. However, the shimming complexity of the system increases with the number of shims required to achieve sufficient spectral resolution per detector. This manuscript addresses this challenge and presents a solution for parallel NMR spectroscopy that integrates a custom-designed probehead with an artificial intelligence (AI)-driven shimming method.

The essential function of an NMR probe implements a sensitive radio-frequency (RF) coil to resonantly detect NMR signals with high spectral resolution. The utilization of stripline coils in microfluidic applications has emerged as a viable solution for high throughput screening and continuous flow measurement^7,8^, particularly in scenarios involving small sample volumes within the micro to nanolitre range. However, their adaptation and operation in parallel, to enable simultaneous analysis of multiple samples through multiple striplines, has remained unexplored. Our custom prototype probehead hardware consists of two parallel and independent channels, each with its own RF coil, flow tube, and shim coil array. The probehead’s design is optimized by finite element simulation (FEM) and implements a new concept of miniaturized stripline as an RF detector, inspired by origami considerations. The topology provides a concentration of the RF magnetic field at the location of the sample, with a reasonable quality factor of $Q=42$ at $650\,\hbox {MHz}$. The probehead also enables sample-centered shimming by utilizing six spherical-harmonics-based (SH) shim lines per channel, which, in contrast to global shimming around a selected isocenter, separately allows each channel to achieve optimal magnetic field homogeneity without affecting the other channel.

Shimming is the process of adjusting the currents in the above-mentioned shim lines to optimize the homogeneity of the magnetic field. However, it is not a trivial task, especially for high-field integrated shim array probeheads that have a large number of shim coils and a complex geometry. No plug-and-play method is available for automated shimming; alternatively, manual shimming can be error-prone and very time-consuming, thereby negating the high throughput advantage altogether. Therefore, a need has arisen for an automated method for shimming parallel NMR channels.

We leverage an artificial intelligence (AI)-driven method that uses deep learning to map 1D-NMR spectra to shim values. Artificial intelligence (AI) covers the development of automated and intelligent systems capable of performing tasks that typically require human intelligence. Deep Learning (DL) is a subset of AI that uses neural networks with multiple layers to learn intrinsic patterns and make predictions from complex data, and it has led to numerous recent advances in the NMR field^9–12^, as summarized^13^. Unlike conventional shimming methods that rely on iterative optimization or predefined shim maps, our method can predict the optimal shim values for each channel with a few random NMR acquisitions, without requiring prior knowledge of the sample or the shim field patterns. Our method uses random sampling to collect training data efficiently, without exponentially increasing the data collection time w.r.t. the number of shims. We also hypothesize that AI can speed up the shimming of parallel NMR channels since the parallel channels show couplings, and in this context, the shim coil sets are mutually non-orthogonal. Our deep neural network (DNN) is trained on a large dataset of experimental shim currents and spectra, and then used to predict the optimal shim corrections for unknown shim distortions.

We demonstrate the performance of our probehead and AI-driven shimming method on a $650\,\hbox {MHz}$ MRI system. We show that our system can achieve narrow spectral linewidths ($<20\,\hbox {Hz}$) without global shimming (only interpolation of linear shims) for both channels simultaneously. Our RF channels can also be operated in broadband mode, e.g., for parallel $^1$H and $^{19}$F measurements. We further show that our AI-driven shimming method can correct large shim distortions, and outperforms the theoretical minimum number of required NMR acquisitions of conventional methods.

In summary, we make the following contributions:

- Design of the first parallel probe for ${650}\,\hbox {MHz}$, with high-order active shims.
- Design of a folded-up stripline with a shim set for ease of manufacturing.
- Implementation of a fast AI-driven shimming method for parallel spectroscopy.

The remaining manuscript is organized as follows: Section “Concept” introduces the design of the custom probehead and its important components, followed by the AI-driven shimming concept. Then, we report promising results in Section “Results”, discuss possible opportunities in Section “Discussion”, and discuss the methods used in Section “Methods”.

## Concept

### Probehead design

**Figure 1:**
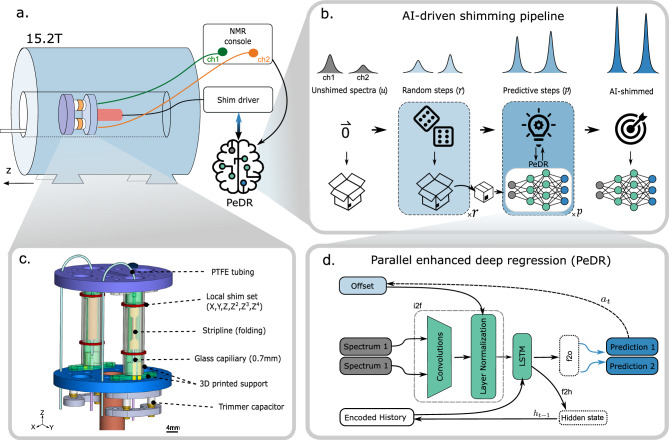
**Concept of parallel NMR and AI-driven shimming.** (**a**) Experimental setup and routing for parallel shimming utilizing a neural network model. (**b**) Parallel enhanced deep regression (PeDR) pipeline with random and predictive shim offset steps. (**c**) Schematic of our custom-built parallel NMR probe targeting a $15.2\,\hbox {T}$ magnet. The main components for each of the two cells include a miniaturized stripline coil, six local shims, and tubing for sample handling. (**d**) Deep neural network architecture (simplified) to predict shim corrections based on 1D-NMR spectra using convolutional and recurrent connections.

We have developed a specially constructed parallel probe for a $15.2\,\hbox {T}$ preclinical MRI magnet (see Fig. 1a), allowing the simultaneous detection of two samples in a synchronized manner. The probe is essential as a conventional single-coil probehead cannot accommodate parallel detection of different samples. Therefore, we employed a new parallel probehead design based on the concept of an NC^3^. The design incorporates several key components, including RF coils (realized by a folded-up stripline), shim coils, and flow channels for sample loading, as depicted in Fig. 1c.

#### Folded-up stripline RF coil

**Figure 2:**
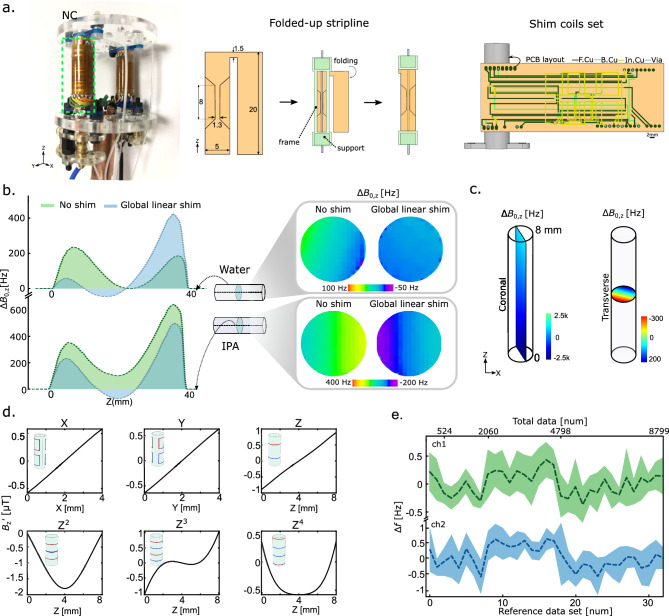
**Hardware development—Parallel stripline resonator with shim coils.** (**a**) A photo of the probe head (left); the schematic step-by-step procedure of folding the flexible stripline coil to a supporting structure (middle); and an exploded view of the shim coils set (right). Abbreviations: F.Cu: front copper layer, B.Cu: bottom copper layer, In.Cu: intermediate copper layer. (**b**) Experimental field maps reveal the insufficiency of global shimming for parallel spectroscopy, considering the non-ideal arrangement of two samples, the intrinsic field distortion, and the susceptibility. (**c**) Simulated $B_{0,z}$ field map of Coronal and Transverse plane across a ($1\,\hbox {mm}$ OD $\times$ $8\,\hbox {mm}$) sample. The simulated distortion represents the susceptibility-induced distortion under a uniform static field ($15.2\,\hbox {T}$). (**d**) Simulated field profiles ($B_z'$) over the sample region of interest generated by local SH shims with $10\,\hbox {mA}$ input, corresponding to the *X*, *Y*, *Z*, $Z^2$, $Z^3$, $Z^4$. The number of turns from top to bottom for each shim is *X*(1,1), *Y*(1,1), *Z*(1,1), $Z^2$(1,2,1), $Z^3$(4,1,1,4), $Z^4$(9,1,1,9). (**e**) Shimming stability of two channels over two days, reported via 160 reference-shimmed spectra that were sampled after intervals of 50 spectra in the datasets.

To achieve parallel detection, we utilized two identically built miniaturized stripline coils as the NMR sensors. However, fabricating a stripline coil as a self-resonant structure presents challenges for miniaturization due to the requirement of a $\lambda$/2 length strip to generate a concentrated RF field at the sensitive regions^14^. Even though additional discrete elements can be added by soldering, paired with a tuning capacitor to form an LC resonance circuit, a bottom-up u-fabrication is still required to provide enough SNR for a mass-limited sample. Also, the metallic via connecting the stripline to the ground plate creates a localized $B_0$ and $B_1$ distortion that can still cause an unwanted shoulder in the NMR spectrum. Figure 2a shows our new stripline coil design based on a folding-up method. The advantage of our design is that we replace the via with an extended arm at the top of the stripline, allowing an all-in-one fabrication procedure for the metal layer, which reduces fabrication complexity for the purpose of miniaturization. The proposed stripline, in Fig. 2a, has an overall length of $25\,\hbox {mm}$, and a width of $5\,\hbox {mm}$, with a sensitive conductor section of $8\,\hbox {mm}\times 1.3\,\hbox {mm}$. The coil features a sensitive volume of interest of $1.57\,\upmu \hbox {L}$ and a self-resonance frequency of $1.75\,\hbox {GHz}$. The coil’s characterization can be found in the Supplementary Fig. S4a,b. The two RF coils were positioned and aligned with the static magnetic field such that their $B_1$ fields are perpendicular to $B_0$. The coils were fabricated on a Polyimide (PI) substrate that offers ample flexibility for folding.

According to the simulation results (see the supplementary information Fig. S4c), the coil exhibits a uniform $B_1$ field, and the effect of localized distortion due to the metal arm is absent at the top of the sample. An experimental impedance measurement shows that the coil has a Q factor of 42 at $650\,\hbox {MHz}$ and an exceptionally high self-resonance frequency of $1.75\,\hbox {GHz}$, which makes it operable in a wide range of magnets. The supplementary Fig. S5 provides a photo of the inner probe and measured S-parameter curves.

#### Shim coils

Each RF coil is equipped with a local shim set (*X*, *Y*, *Z*, $Z^2$, $Z^3$, and $Z^4$) to correct local field distortions, as global shims are not sufficient for shimming multiple samples for parallel detection.

We experimentally verified the need for localized shim sets by placing two high aspect-ratio samples (water and isopropanol) in the magnet’s isocenter to map their spatial field distortion. The details of the experimental setup are reported in Section “Methods”. We can see from the central axial slice in Fig. 2b that both samples have different $\Delta B_0$ before shimming. The $B_0$ inhomogeneity reduced after the automatic linear shim in the case of the water sample, but it did not improve for isopropanol. This was caused by the automatic linear shim algorithm of the spectrometer, which optimized the signal with the highest intensity. Also, the global shims can only be optimized relative to one isocenter. This demonstrates that the global shims are insufficient for shimming multiple samples for parallel detection. Further experimental evidence is provided in the supplementary material, namely in the supplementary Fig. S1, showing that only a single channel can be shimmed with global shims.

To develop a practical arrangement of shims to fit on a single 3-layer flexible PCB and effectively address the majority of $B_0$ inhomogeneities while minimizing the number of required shims, we conducted FEM simulations of the NC to access the $B_0$ field distortion within a $1\,\hbox {mm}\times 8\,\hbox {mm}$ region of interest. Field distortions depend on the sample’s geometry in the sensitive region. Commercial shim systems utilize more than 20 SH shims^15^ to reach an acceptable linewidth for different sample shapes to acquire high-resolution NMR spectra. While the direct adoption of 20 coils for localized shimming brings complexity to the probe, which exacerbates as the number of cells increases, a smarter shim-set design that only considers the most effective gradients becomes essential. For example, a very high aspect ratio of sample volume is a 2D analogue of a wire, for which we would expect that the inhomogeneity induced by high-order SH functions on a coronal slice should be negligible. This reduces the necessary amount of shim coils needed for parallel shim arrays. We executed $B_0$ simulations with COMSOL, as described in Section “Methods”, showing that the majority of inhomogeneity is along the z-direction, with up to $3000\,\hbox {Hz}$ field differences, as seen in Fig. 2c. At the same time, the magnetic field is mainly linearly distorted by $500\,\hbox {Hz}$ on a cross-sectional x-y plane. Both the experimental and simulated field on the x-y plane show a linear distortion pattern (see Fig.2c,e), indicating that the primary source of distortion originates from the spherical harmonics (SH) of *X*, *Y*, *Z*. Furthermore, simulations along the z-direction show distortions of higher-order zonal functions. Based on the above results, we designed the shim set, ensuring it could be integrated into a single 3-layer PCB and possess sufficient shimming capabilities. The shimming profiles $B_z'$ are illustrated in Fig. 2d.

A shimming stability test of the rolled-up shim set (Fig. 2e) connected to a custom-built shim driver (see Section “Methods”) indicates that both shimming channels possess a stable linewidth. In our four data subsets collected over two days, and a reference spectrum measured every 50 random acquisitions, we gained 160 reference-shimmed spectra. Their linewidths only fluctuate within the range of $\pm 1\,\hbox {Hz}$, calculated over blocks of 5 spectra.

#### Flow channels

The custom NMR parallel probe also features two inlet and two outlet ports for fluidic tubing, allowing continuous high-throughput screening measurements^1^. The sample inlet and outlet ports are connected to the top and bottom of the NC through Polytetrafluoroethylene (PTFE) tubing securely connected to a pre-insert sample-handling glass capillary to prevent background signals from the tubing. An exploded view can be seen in Fig. 2b. The samples are transferred by a syringe pump to the sensitive region of the stripline coil through the PTFE tube.

### AI concept

Shimming becomes a challenging task in parallel spectroscopy, where multiple channels are used to acquire signals from different regions of interest. Unlike single-channel spectroscopy, where a single set of orthogonal shim coils can be used to correct the field inhomogeneity, parallel shimming requires a more sophisticated approach that considers shim interactions and RF couplings between the channels. Moreover, our custom hardware shows non-idealities due to manual assembly, resulting in non-orthogonal shim fields that complicate the optimization problem. Even though the shim coils may be orthogonal for each channel taken separately, they are not necessarily simultaneously so for two or more coils, leaving classical algorithms such as the simplex prone to perform many redundant actions. We study shimming supported by artificial intelligence, i.e., AI-driven shimming. We argue that AI can handle the high-dimensional and non-linear nature of the shimming problem, and can learn from the complex cross-sensitivity among the channels. We also show that AI can adapt to the non-orthogonal shim fields and find good shim settings for each channel.

#### Shimming process

We adopt a previously reported AI-driven shimming process^16^, which consists of an initialization step, two phases with varying steps, and a final wrap-up step (see Fig. 1b). After acquiring the initial, unshimmed spectrum *u*, a fixed number of *r* random shim offset steps are applied to create a model-internal shimming history and aid the DL model to orientate itself in shim space. Then, *p* predictive steps are applied, where the model’s output serves at the next shim action $a_t$. Finally, we observe a shimmed spectrum $u(a_t)$ after $t=r+p+1$ steps.

During random steps, $a_{t}$ is Gaussian noise $\mathcal {N}$, and during prediction steps, the last prediction ($a_{t}= \hat{\textbf{y}}_{t-1}$) is used to generate the next spectrum.

#### Architecture

Our neural network architecture for AI-driven shimming incorporates convolutional layers^17^, a long short-term memory (LSTM) layer^18^, fully connected (FC) layers, dropout regularization, layer normalization, ReLU^19^ and tanh activations. These components collectively enable efficient feature extraction, temporal modelling, non-linear relationship learning, regularization, and improved training stability, respectively. By integrating these elements, our architecture aims to effectively process input data and make accurate predictions in the context of shimming for NMR applications.

We follow a similar architecture for AI-driven shimming^16^ but extend it to handle the parallel scenario. The model (see Fig. 1d) consists of (1) a convolutional part that extracts features from the two input spectra at each time step *t*, (2) a fusion layer that incorporates the past actions $a_{t}$ w.r.t. the initial unshimmed spectrum, (3) a recurrent cell (LSTM) that allows learning temporal dependencies in shimming sequences of flexible lengths, and (4) an output head (f2o) that predicts our shim corrections.

In detail, the i2f section includes a convolutional block and a fusion layer including normalization. The convolutional block consists of 3 layers, each represented by a sequence of convolutions (64 filters, kernel size 41, stride 2), ReLU activation, dropout, and pooling. The fusion of convolutional feature maps and last actions is done by concatenation and layer normalization. These features are fed into the LSTM cell with the last hidden state $h_{t-1}$ to generate a new set of features and the next hidden state. The prediction is made by f2o, whose first layer normalizes the features, and then feeds them through one FC layer with dropout and ReLU, and a final FC layer with tanh activation.

**Figure 3:**
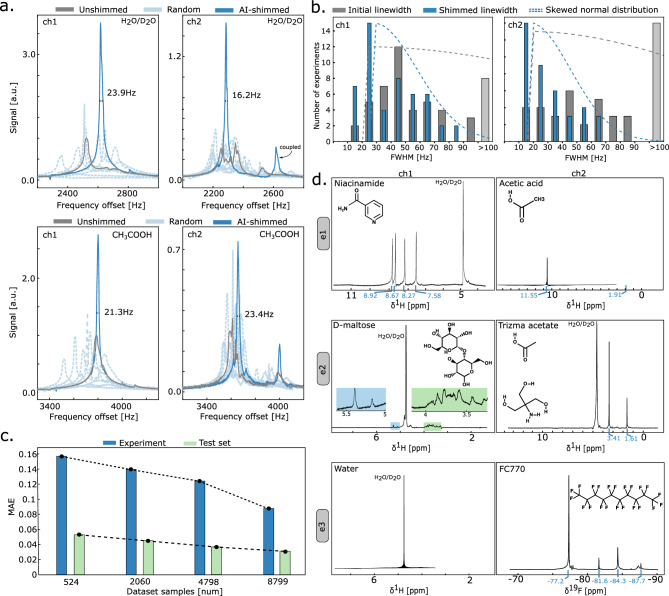
**Proof-of-concept using AI-driven shimming for two parallel channels.** (**a**) Exemplary results of AI-driven shimming on water (top) and acetic acid (bottom). (**b**) Linewidth distribution (histogram and fitted skewed normal distribution) before and after AI-driven shimming. (**c**) Influence of dataset size on the prediction performance, reported on the test set and experiments. (**d**) NMR spectra recorded with synchronized excitation and reception acquired in the two-channel probehead. (**e1**) $^1$H NMR spectra of niacinamide and acetic acid. (**e2**) $^1$H NMR spectra of D-maltose and trizma acetate. (**e3**) $^1$H NMR spectrum of 50 $\%$ (v/v) $\textrm{H}_{2}\textrm{O}$/$\textrm{D}_{2}\textrm{O}$ and $^{19}$F NMR spectrum of FC770.

## Results

### Parallel spectroscopy with custom probehead

The parallel 1D $^1$H NMR homonuclear (experiments e1 and e2) spectra of two samples are presented in Fig. 3d measured in parallel by two stripline coils: (e1), where channel 1 is filled with a ${0.4}\,\hbox {M}$ Niacinamide solution and channel 2 is filled with ${17.4}\,\hbox {M}$ Acetic acid, and (e2), where channel 1 is a ${0.16}\,\hbox {M}$ D-(+)-maltose solution and channel 2 is a ${0.3}\,\hbox {M}$ Tris(hydroxymethyl)aminomethane acetate (Trizma) solution. As demonstrated in the figure, each proton is labelled with respect to its chemical shift. The spectra are averaged from 256 scans. Due to the RF coupling between the two RF channels, mainly caused by the RF coils and coaxial cables, we post-processed the two raw spectra by signal subtraction. All the necessary main peaks are resolved in the measurement results.

While homonuclear parallel NMR offers the advantage of directly comparing signals from similar samples, it introduces the issue of signal coupling between the two channels. This occurs because both coils simultaneously excite and receive signals at the same frequency. This strong coupling can be minimized using a time-interleaved^5^ pulse sequence; however, this is against our concept of true synchronization. Here, we tested the probe in heteronuclear mode, performing parallel spectroscopy simultaneously at different striplines, one tuned to $^1$H , and the other to $^{19}$F . We transferred two samples of water and FC-770 ($\textrm{C}_{10}\textrm{F}_{22}$) and measured them accordingly (see Fig. 3d experiment e3).

### AI-driven shimming for parallel spectrosopy

#### Spectral quality and performance metrics

To assess the performance of our AI-driven shimming method, we report the linewidth at the full width at half maximum (FWHM, $50\%$), where the linewidth is measured on a Voigt line fit^20^ if the peak shows splitting. Furthermore, we report the mean absolute error (MAE) and custom metrics: the direction ratio $\text {DiR}\in [0,1]$ that indicates whether the predictions’ and targets’ signs match^21^; and the success rate $\text {SR}\in [0,1]$, which is 1 for a single experiment, if the spectral peak intensity increased for both channels.

#### Evaluation results

We evaluate our neural network model, which was trained on previously acquired real data according to the scheme, as reported in Section “Methods”.

Our AI-driven shimming method PeDR can successfully and simultaneously shim distorted spectra from two channels with six shims each to well-defined lineshapes, within only 10 NMR acquisitions. Evaluated over 50 random distortions $\in 2\times$ the reference values, we can shim from $93 \pm 142$ $\hbox {Hz}$ for channel 1 and $91 \pm 102$ $\hbox {Hz}$ for channel 2 to $39 \pm 19$ $\hbox {Hz}$ and $26 \pm 20$ $\hbox {Hz}$, respectively. This corresponds to a relative linewidth improvement of $+139\%$ and $+436\%$, drastically reducing the standard deviation. Figure 3b shows a clear trend in the distributions between unshimmed and shimmed linewidths for our experiments. Qualitatively speaking, we achieve an 88% success rate, a DiR of 0.9, and a total MAE between real distortions and predictions of 0.089.

Figure 3a visualizes the process of our method: Starting from the unshimmed spectrum (grey), we apply random offsets. The corresponding spectra (light-blue), are fed into our model to predict a shim correction term, which yields shimmed spectra after a number of predictive steps (blue). Note here that the influence of random steps is more crucial than predictive steps, which already have been discovered^16^.

To study the generalization ability of our DL method, we successfully demonstrate shimming on acetic acid (peak X-OH). We achieve a 78% success rate, DiR of 0.85, and an MAE of 0.112, corresponding to a slight drop in performance. One reason is that the dataset consists of HDO spectra only, leaving the model uncertain in its predictions. Nevertheless, we argue that our model (with a more diverse dataset) can be applied to any single peak in the spectrum, i.e., a reference TSP peak.

##### Influence of dataset size

While it is known that large datasets are required for achieving high performance in deep learning models, the exact relationship between dataset size and performance remains unclear, especially for NMR applications, where data acquisition is expensive. Thus, we tested our methods trained on subsets of the whole dataset. Unsurprisingly, using more data during training reduces the error on both the test set and in experiments (see Fig. 3c). We further observe a discrepancy between offline and online evaluations, which could be caused by residual stochasticity of the data generation process, or an environmentally-caused drift of the experimental setup, which could be tackled through data harmonization^22^.

##### Theoretical comparison of AI-driven shimming to traditional methods

One of the main advantages of our AI-driven shimming method is that it can achieve decently shimmed spectra with a minimal number of acquisitions. In contrast, traditional methods are generally slow and require initial acquisitions to initialize the optimization algorithms. However, the implementation and interface of these methods do not exist, so we can only compare our method to the theoretical minimum number of acquisitions required to initialize them.

Shimming based on parabolic interpolation^15,23^, referred to as “parabola”, fits a quadratic function to three points and finds the minimum of the function. The simplex algorithm^24,25^ uses a geometric polytope (a “simplex”) of $n+1$ vertexes, where each vertex is represented by the quality criterion corresponding to specific shim settings. The simplex then evolves through shim space using geometrical operations (reflection, expansion, contraction) until a local minimum is found. Both algorithms require an initial set of points (or brackets) to start the optimization. The original simplex algorithm converges as $2^{\text {polynomial}(n)}$, where *n* is the number of iterations. Successive parabolic interpolation has a superlinear convergence rate of 1.325, but is prone to get stuck when points are colinear. More robust methods are available but require the evaluation of derivative functions.

The theoretical minimum number of acquisitions for these methods and *m* parallel coils is as follows. Parabola initialization requires $m\cdot n \cdot 3$ acquisitions. With optimal brackets and the best shim value lying at the parabola’s minimum, 1 acquisition per shim coil is required to check the resulting linewidth. In our case, this would sum up to 48 acquisitions. Simplex initialization needs $(m\cdot n)+1$ spectra, and then each optimization step 2.5 acquisitions, on average. As the simplex method is known to converge slowly^26^, e.g. up to 90 steps for a single channel and 4 spectra^16^, more than 200 additional acquisitions would be necessary. In conclusion, the simplex definitely takes more than 13 acquisitions, without any optimization step for our scenario.

We demonstrated that we are below that threshold, and can already predict shim corrections near the global minimum region.

As a side note, manual shimming to the reference values took approximately two hours, which shows how impractical this approach is for parallel spectroscopy, not only because it is inherently slow, but because it does not scale with increased parallel sites.

## Discussion

In this section, we discuss several limiting design choices of our study, including the number of RF channels and shim lines, the inclusion of global shims, DL dataset and algorithmic considerations, and RF couplings.

In general, RF coils could also be used to circumvent $B_0$ inhomogeneities^27^. However, gradients, especially shielded gradients, are required to perform RF/pulse shimming, which are difficult to miniaturize for parallel hardware.

Our probe head prototype has only two channels, considering the system complexity and bore space to handle cables and shielded shim lines. Furthermore, we only incorporate six shim coils per channel for easy integration on a single 3-layer PCB. The wrapped local shim set utilized in the study is highly stable for a long-time experiment over two days with negligible linewidth fluctuation. Also, our simulations experiments show that our chosen local shim set reduces the major field inhomogeneities without employing high order ($>1$) global shim coils. The system’s global shim coils are only incorporated to interpolate the iso-centre between the channels. However, a combined shimming approach should enhance linewidths.

Our experiments specifically concentrated on conducting homonuclear NMR experiments on each sample, because they provide an effective assessment of both $B_0$ homogeneity and, more significantly, RF coupling. Even though we have demonstrated that the system allows performing heteronuclear experiments at different sites, which favours multinuclear experiments and potentially allows for multiplexing NMR experiments by direct detection of different nuclei in a single experiment^28^.

Using a water-only dataset with 8k samples in our study allows for a focused evaluation of our AI-driven shimming approach, providing a controlled environment to assess its shimming effectiveness. We can thoroughly investigate the impact of our methodology without the complexities introduced by other sample types. Furthermore, the relatively small dataset size minimizes resource requirements, such as expensive data collection, while still allowing investigation of model behaviour and limitations. Future research can expand the dataset for broader generalization.

Despite being much faster than traditional methods, our DL algorithm does not guarantee convergence to optimum shim values due to the general stochastic nature of DL. A feedback loop inside AI-driven shimming algorithms could compensate for this issue.

Finally, we observed the coupled components in our spectra due to the inter-channel RF coupling. These components could be removed by parallel channel signal decomposition.

## Methods

### Finite element method

The geometry of the stripline coil was modelled in CAD software (Solidworks 2014, Dassault Systemes S.A.) and imported to a FEM simulation environment (COMSOL Multiphysics 5.4, DC and RF modules, COMSOL AB, Sweden). The static magnetic response of the NC to $B_0$ field, to reveal the $B_0$ homogeneity, was simulated with an imposed magnetic flux density of $15.2\,\hbox {T}$, with an error tolerance of $10^{-3}$ parts per billion and 199k mesh elements. The electromagnetic simulation to reveal the $B_1$ distribution was performed using a lumped element model, with a port input of $1\,\hbox {A}$. For the shim coil simulation, all looped coils were excited with $10\,\hbox {mA}$ input, since the field strength scales linearly with the current.

### $B_0$ field map sequence

Sample-dependant $B_0$ inhomogeneities in the parallel NMR experiments were demonstrated with the following $B_0$ map experiments. The experiments were performed on a $15.2\,\hbox {T}$ ultra high field magnet (Bruker, Ettlingen, Germany) with a commercial probe equipped with a $35\,\hbox {mm}$ diameter birdcage coil tuned to ${650}\,\hbox {MHz}$ for $^1$H nuclei. Water and isopropanol were used as samples inside a test tube of ${2.4}\,\hbox {mm}$ inner diameter, and placed off the centre of the z-axis at a ${30}\,\hbox {mm}$ distance to each other. $B_0$ map experiments were carried out with a standard FieldMap sequence available within the Paravision (Bruker, Ettlingen, Germany) software. We obtained a field map with a spatial resolution of $64\times 64$ pixels for 200 axial slices, over a field of view of $10\times {10}\,\hbox {mm}^2$, a repetition time of $35\,\hbox {ms}$ , and an acquisition time of ${7}\,\hbox {min}$ ${28}\,\hbox {s}$ for each experiment. The $B_0$ map of the central axial slice of the two samples (${2.4}\,\hbox {mm}\, \text {ID} \times 40\,\hbox {mm}$, aspect ratio = 16.6) before and after automatic linear shimming are shown in Fig. 2c. The mean $\Delta B_{0z}$ of each axial slice is calculated and plotted for the 200 slices, which shows the coronal $B_0$ field variation with a characteristic higher order $B_0$ inhomogeneity.

### Probehead manufacturing

The parallel probe head (depicted in Figs. 1c and 2a) was manufactured as follows. The flexible PCB, forming the shim and RF coils, was ordered from a vendor (multiPCB, Germany). Both the RF coil and shim coil have a thickness of $18\,\upmu\hbox {m}$ of copper on a $25\,\upmu\hbox {m}$ of Polyimide (PI) substrate, and ${25}\,\upmu\hbox {m}$ of PI cover layer. Note that the shim set was made in three layers to avoid overlapping between shims and overcrowding the layout with vias. The RF coil was folded on a 3D-printed support structure aligned with a pre-designed frame, with ${0.8}\,\hbox {mm}$ thickness, and a pre-inserted sample-handling glass capillary (${700}\,\upmu\hbox {m}$ OD, VitroTubes, VitroCom) to avoid susceptibility artefacts. A photo of the coil can be seen in Fig. 2a. Both coils were attached to supporting structures using instant glue (UHU Plus) and soldered to the tune-and-match circuit, with high Q non-magnetic trimmer capacitors (Voltronics, V9000). All the supporting structures were 3D-printed in-house (PRUSA).

### Custom-built constant shim drivers

For a precise and extremely-low drift homogenizing of the $B_0$ field, a custom 28 channel ${\pm 300}\,\hbox {mA}$ shim current source was developed (see the supplementary Fig. S3). Each channel was designed as a cascade amplifier stage^29^ in which an input control voltage from a commercial 16-bit multi-channel USB digital-to-analogue converter (DAC) card was converted to an output current of ${\pm 300}\,\hbox {mA}$ with a set current step of approximately ${9.2}\,\upmu \hbox {A}$. Very low drift, rather than absolute current accuracy, was the focus of the design. The scheme explaining the principle of one channel can be seen in the supplementary Fig. S2. The $\pm 10\,\hbox {V}$ output of the DAC channel was divided by 10 via the 10 ppm/$^\circ C$ drift precision resistors R1 and R2 and fed to the input of the zero drift op amp OP1 with an internal offset voltage drift of only about $36\,\hbox {nV}$/$^\circ C$. OP2 acts as a power amplifier stage with a voltage gain of 2. This power amplifier heats up during operation and will change its offset voltage and input bias currents. R8 closes a feedback loop to OP1 so that, in combination with OP2, it can set the output current. In closed-loop operation, the voltage drop across the current sense resistors R4 and R6 equals the voltage drop across R2. The series-connected R4 and R6 are ${3}\,\hbox {W}$ power resistors with a maximum drift of 60 ppm/$^\circ C$, dissipating only a maximum of ${135}\,\hbox {mW}$ each, and were thermally coupled to compensate for the temperature of their internal thermal junction voltages, referring to this application note^30^. C1, C2, C9, R5, R3, and R8 are for OPV input bias current compensation, and limit the circuit bandwidth for high-frequency noise suppression, allowing around ${20}\,\hbox {ms}$ current settling time. Low ohmic resistors were used at the circuit’s input to reduce thermal noise.

The copper layers of the PCB have an additional voltage drop and temperature gradients, especially when carrying high currents. The layout was designed to thermally isolate the channels from each other. The main ground was designed in a star configuration from a low impedance centre, which carries the DAC card, and in 3 vertical parallel-connected ground plane structures of the 4-layer PCB. This minimized voltage drop across the ground planes and crosstalk between channels. In the mechanical design, the power OPVs were placed outside the PCB surface on separate heat sinks, and the current sensing resistors were placed in the free space above the PCBs in a continuous airflow. Due to the large ground copper layers, the PCBs remained in cold conditions with low thermal gradients along their surface. Special low-output noise 5V switching power supplies were used, and additional EMI noise filters were implemented on the circuit boards.

### Formal deep learning problem definition for parallel AI-driven shimming

Let $\mathcal {D}=\{(\textbf{x},\textbf{y})_{i}\}_{i=1}^{|\mathcal {D}|}$ be our static dataset, where $(\textbf{x},\textbf{y})_{i}$ is an input-output pair. With *m* being the number of parallel channels, and *n* the number of separate shim coils, the input sequences $\textbf{x} \in \mathbb {R}^{m\times t \times (L,n)}$ of *t* entities are defined as $\textbf{x}_m=\left[ \big ( u_m(\vec {0}),\vec {0} \big ) , \big (u_m(a_1),a_1 \big ),..., \big ( u_m(a_t),a_t \big ) \right]$, where the unshimmed spectrum $u_m$ of length *L* for each channel *m* changes as a function of (random) shim offsets (or actions) $a\in \mathbb {R}^{n}$. Each associated target $\textbf{y} = (y_1,y_2,...,y_{m\cdot n}) \in \mathbb {R}^{m\cdot n}$ represents the distortion from the reference shim values and is defined as a real-valued vector of $m\cdot n$ elements. The regression model $\texttt{F}_{\mathbf {\theta }} (\cdot )$, represented by a custom deep neural network with parameters $\mathbf {\theta }$, predicts the shim correction terms $\hat{\textbf{y}} = \texttt{F}_{\mathbf {\theta }} (\textbf{x})$, such that $\textbf{y}_i-\hat{\textbf{y}}_i \approx 0$. The network parameters $\mathbf {\theta }$ are learned in a supervised manner using the dataset $\mathcal {D}$ to minimize the loss term $\mathcal {L}$ between the prediction $\hat{\textbf{y}}$ and the target $\textbf{y}$. Note that the shim distortions *S* w.r.t. the reference spectrum serve as the labels $\textbf{y}$, whereas the shim offsets (or actions) w.r.t. the first, unshimmed spectrum are denoted as $a_t$.

### Dataset collection

Machine-specific hardware non-idealities require the collection of a real dataset, which allows AI to learn specific features for shimming. Thus, we acquire a real dataset with our custom probehead with 8799 samples. Each sample contains two spectra as a result of random shim distortions *S* to the reference shim values Ref (as reached by manual shimming), following a Gaussian distribution with $\sigma = \frac{1}{3} \times \texttt {Ref}$. The global linear shim values are interpolated between the optimal shim settings between the two NC, as achieved by the manufactures automated shimming algorithm for each channel. Detailed parameters are reported in the supplementary materials.

Based on the collected dataset, we construct our train, validation, and test set for DL training with ratios $80/10/10\,\%$, respectively. Our sequences are constructed online, at random, and new for each individual target value. This means that during training, all steps are assumed to be random offsets from the initial, unshimmed spectrum *u*(0), which we mine from the subsets respectively.

### Deep learning training of the PeDR model

Training of the neural network is performed with similar settings as Becker et al.^16^, and described in detail below.

#### Normalization

All spectra are cut to a region of interest (ROI) of size 4096, and then downsampled by a factor of 2. The first two spectra of a sequence $\textbf{x}$ are normalized to [0, 1] for the spectrum with the highest intensity, and the following spectra according to the first spectrum’s maximum. The regression targets, i.e. the shim correction values, are normalized to $[-1,1]$.

#### Augmentation

We utilize data augmentation to artificially increase the amount of data and variance. We perform uniformly random $Z_0$ shift $\in [-4,4]$, uniform label noise of 0.1, uniform shim interaction noise of 0.1, first-order phase distortions of ${\pm 0.5}$, and additive white Gaussian noise (AWGN) with a signal-to-noise ratio (SNR) of 30.

#### Training

We train our model using the Pytorch framework^31^ for 100 epochs with a learning rate of $1\times 10^{-4}$ (reduced on plateau by an automated scheduler), a batch size 256 and the Adam optimizer^32^ to minimize the Huber loss. The sequence (or shim trajectory) length during training is increased by 2 for every 25 epochs, ranging from 4 to 10. Our model achieves a normalized MAE ($\in [0,1]$) of 0.033 on the test set of our static dataset $\mathcal {D}$.

#### Hardware requirements

We performed DL training with an AMD Ryzen Threadripper 3970X equipped with $256\,\hbox {GB}$ RAM, and two graphics processing units NVIDIA GeForce RTX A5000. The dataset roughly allocates ${4.8}\,\hbox {GB}$ of disc space.

#### Hardware interface

 Dataset collection and DL evaluation require automated execution of different scripts or programs on varying operating systems. We utilised a Python click bot to control all relevant interfaces easily.

### Supplementary Information

Supplementary Figures.

## Data Availability

The raw data generated and analysed during the current study is available upon request. The dataset is publicly available via https://github.com/mobecks/ShimDB and this repository^33^.

## Code availability

The deep learning code is publicly available in the repository https://github.com/mobecks/parallel-ai-shim.
