# Supplementary material for: Artificial intelligence-driven shimming for parallel high field nuclear magnetic resonance
Source: Sci Rep. 2023 Oct 20;13:17983. doi: 10.1038/s41598-023-45021-6 (PMC10589267; doi:10.1038/s41598-023-45021-6)
Supplement: Supplementary file 1 — Supplementary Figures. [file 41598_2023_45021_MOESM1_ESM.pdf]

Supplementary Materials for parallel shimming

Moritz Becker†, Yen-Tse Cheng†, Achim Voigt, Ajmal Chenakkara, Mengjia He, Sören Lehmkuhl, Mazin Jouda⋆, and Jan G. Korvink⋆

†Contributed equally.

⋆Corresponding author. Email: mazin.jouda@kit.edu, jan.korvink@kit.edu,

1 Global vs local shimming

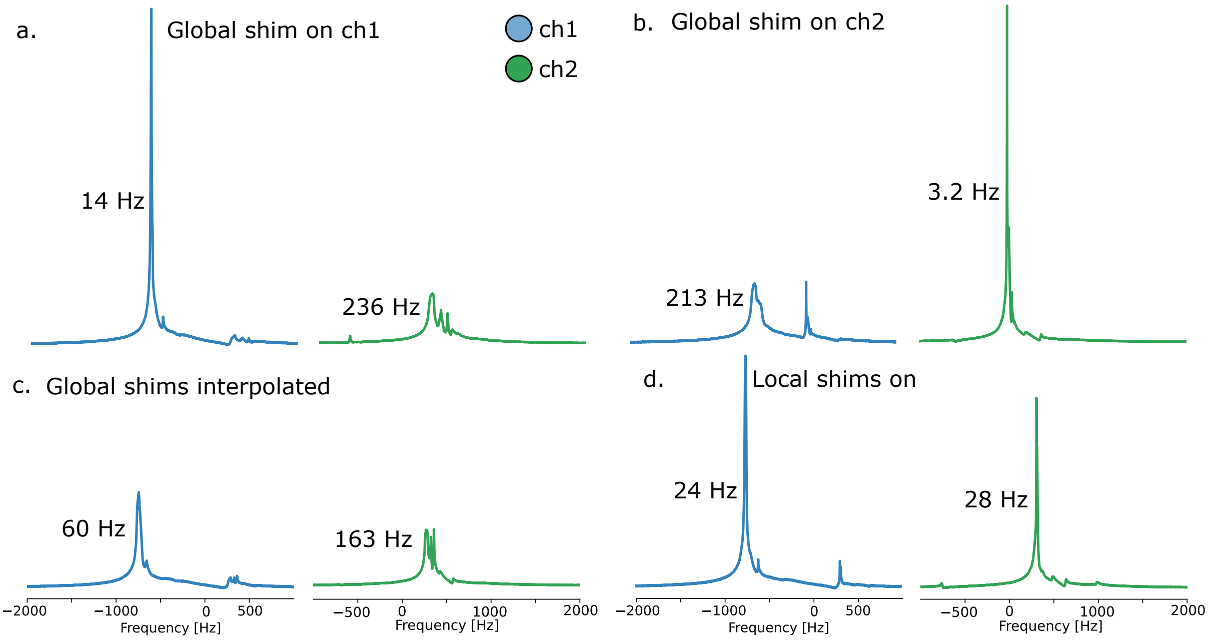

Figure S1: Global vs local shimming. (a)+(b) Global automatic shimming of a single channel will leave the other channels with lousy linewidths. (c) Interpolating the optimum linear shims will reduce both peaks' linewidths but not to spectroscopic resolution. (d) After mild (quick testing) local shimming, both channels show decent linewidths. The linewidths are measured on a Voigt-fit if the peak shows splitting. Both channels are filled with pure H_2O for comparison.

2 Development of 28Ch shim drivers

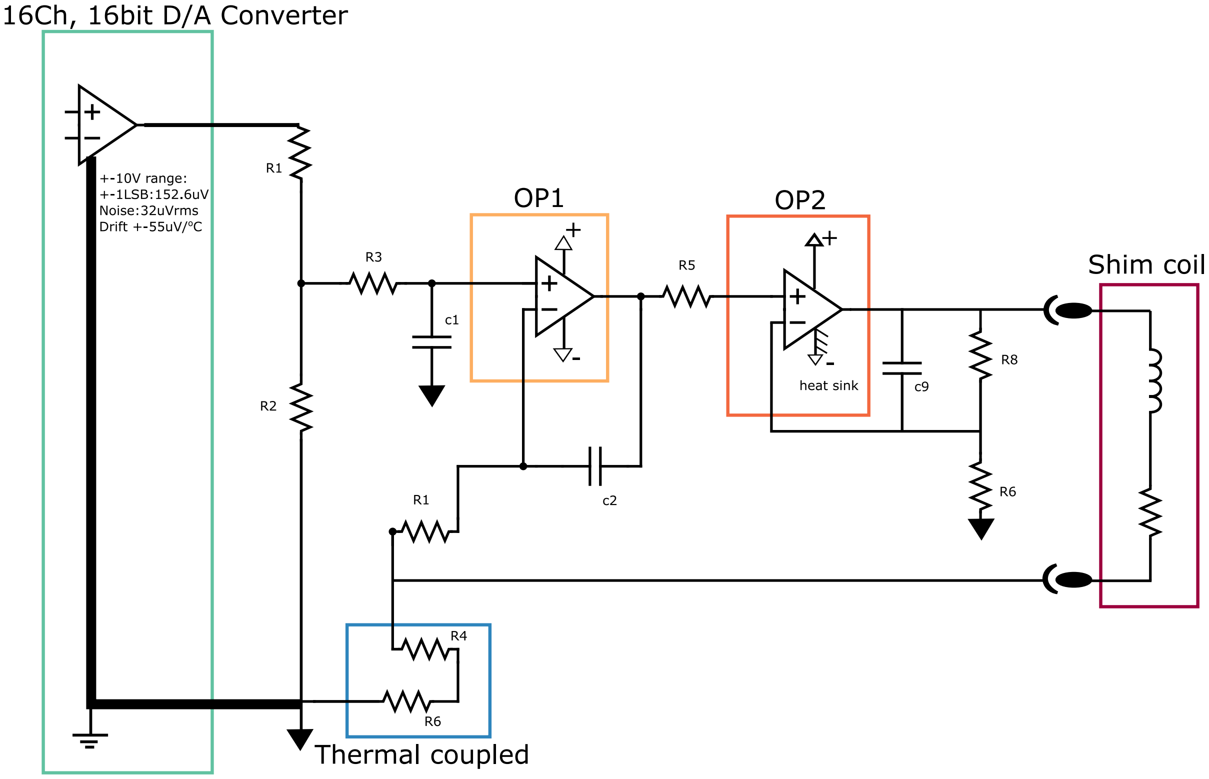

Figure S2: Single-channel DC current generation based on cascaded operational amplifiers.

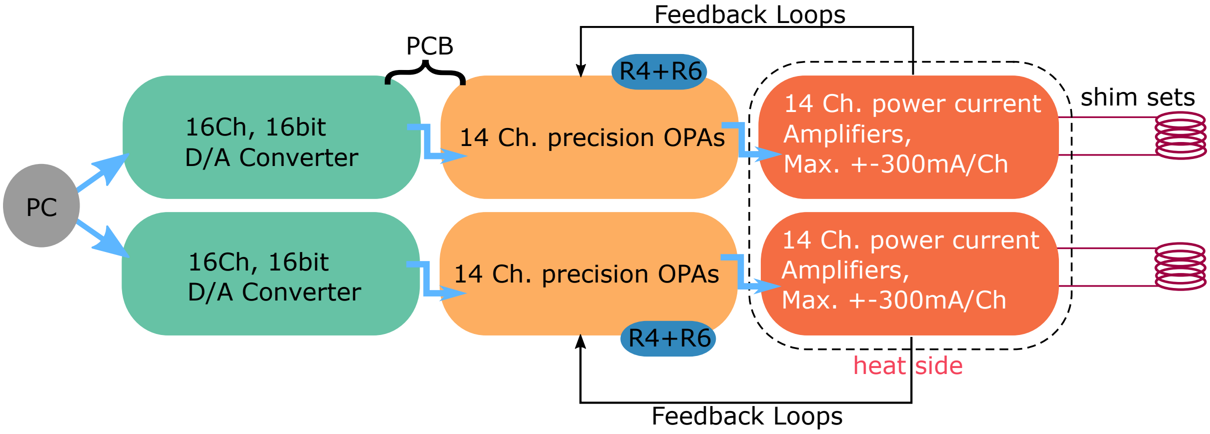

Figure S3: Schematic of the 28 channel shim current source.

3 Probehead and RF simulation for stripline coil

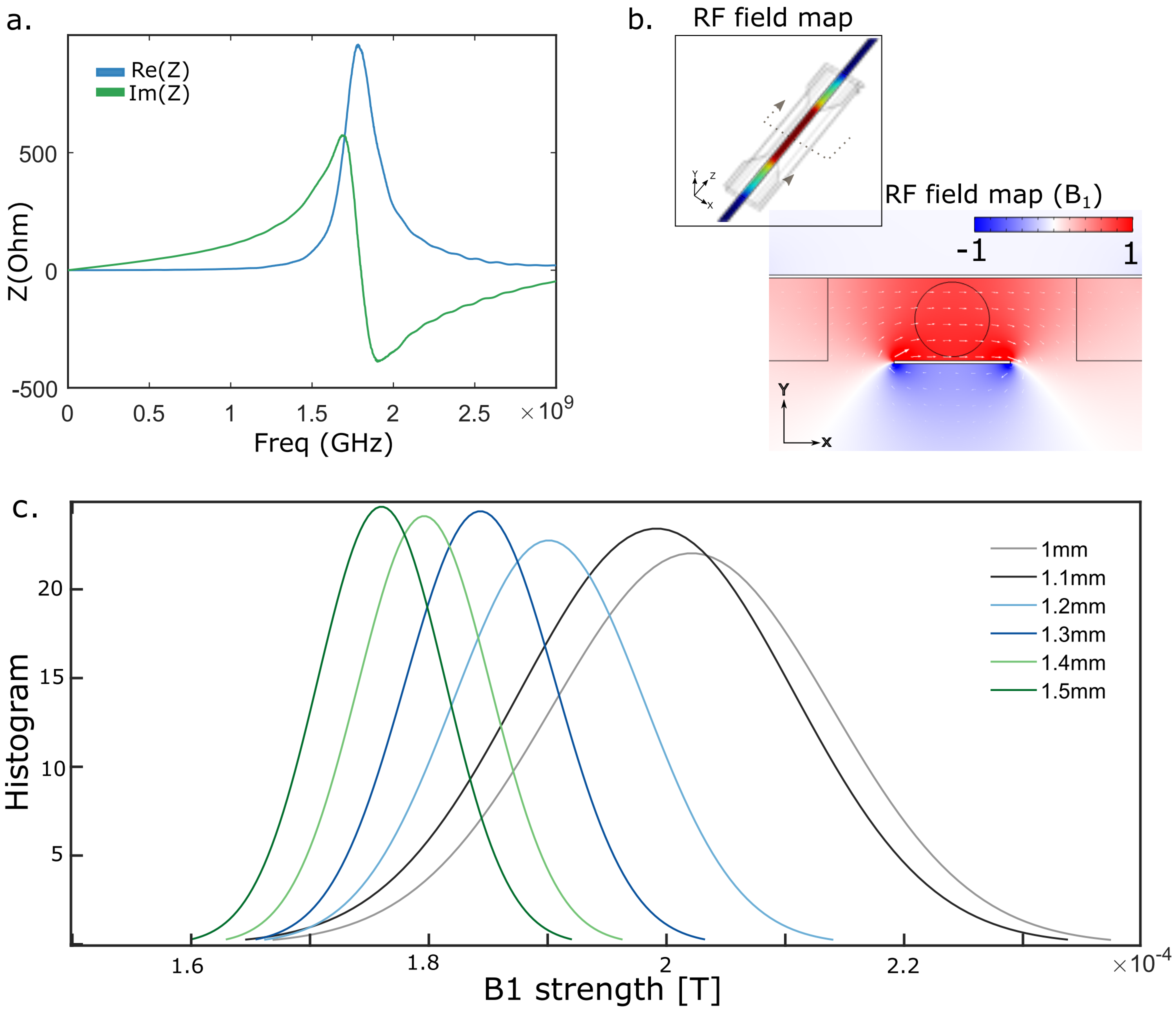

Figure S4: (a) Measured impedance by network analyzer for the fold-up stripline coil, displaying the self-resonance frequency at 1.75 GHz, with a Q factor of 42 at 650 MHz. (b) Comsol simulation result of the stripline's radiofrequency field map at 650 MHz over the central cross-section. The sample capillary is indicated as a black circle, placed where the radiofrequency field is most uniform. (c) Simulated B_1 field uniformity for different stripline widths. The curves depict a histogram showcasing the distribution of B_1 field intensities across the entire sample volume. The simulation of B_1 is conducted using the RF module in COMSOL Multiphysics.

4 Manufactured probehead

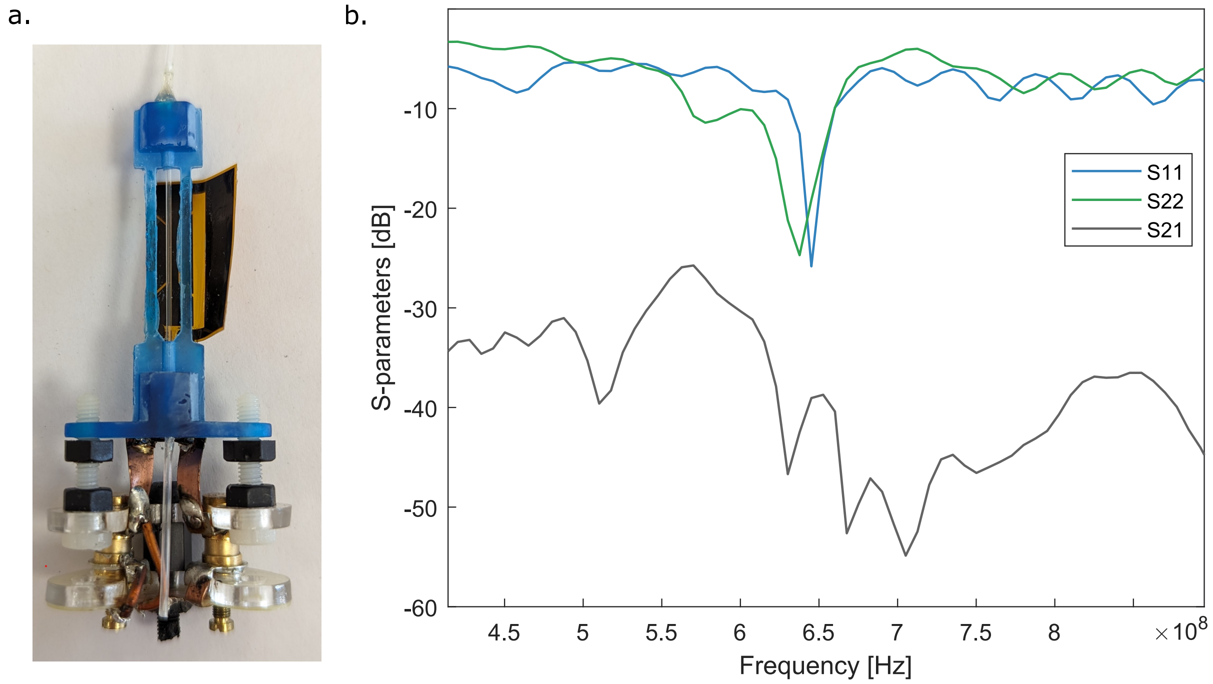

Figure S5: (a) Picture of inner probe. (Note: The stripline is unrolled to show the inner printed structure.) (b) Experimental S-parameter curves for both RF channels, including cables.

5 Deep learning details

5.1 Dataset parameters

Dataset parameters	NC channels	2
	Local shim coils	6
	Reference values `Ref`	$[0, 90, 140, 14, 0, 60, 8, 80, 20, 22, 0, 0]$
	Shim range R	$\pm 2 \times$ `Ref`
	Sample 1 and 2	50 vol% H_2O in D_2O
	Number of spectra per subset	$\{524, 1536, 2738, 4001\} \times 2$
Acquisition parameters	Nucleus	^{1}H
	Excitation bandwidth	5 kHz
	Recorded FID points	19736
	Repetition time	1500 ms
	Postprocessing	Bruker default

Table S1: Parameters of the parallel randomized shimming dataset (PaRandShimDB).
